# The Evaluation, Diagnosis, and Management of Infantile Hemangiomas—A Comprehensive Review

**DOI:** 10.3390/jcm14020425

**Published:** 2025-01-10

**Authors:** Arnes Rešić, Zoran Barčot, Dubravko Habek, Zenon Pogorelić, Marko Bašković

**Affiliations:** 1 Department of Pediatrics, Children’s Hospital Zagreb, Ulica Vjekoslava Klaića 16, 10000 Zagreb, Croatia; 2 University Department of Health Studies, University of Split, Ruđera Boškovića 35, 21000 Split, Croatia; 3 Department of Pediatric Surgery, Children’s Hospital Zagreb, Ulica Vjekoslava Klaića 16, 10000 Zagreb, Croatia; 4 School of Medicine, University of Zagreb, Šalata 3, 10000 Zagreb, Croatia; 5 Croatian Academy of Medical Sciences, Kaptol 15, 10000 Zagreb, Croatia; 6 Department of Obstetrics and Gynecology, Clinical Hospital Merkur, Zajčeva ulica 19, 10000 Zagreb, Croatia; 7 School of Medicine, Catholic University of Croatia, Ilica 242, 10000 Zagreb, Croatia; 8 Department of Pediatric Surgery, University Hospital of Split, Spinčićeva ulica 1, 21000 Split, Croatia; 9 School of Medicine, University of Split, Šoltanska ulica 2a, 21000 Split, Croatia; 10 Scientific Centre of Excellence for Reproductive and Regenerative Medicine, School of Medicine, University of Zagreb, Šalata 3, 10000 Zagreb, Croatia

**Keywords:** infantile hemangioma, vascular tumor, β-blockers, propranolol, timolol, laser therapy, surgery, infant, children

## Abstract

Infantile hemangioma (IH) is the most common pediatric benign vascular tumor. Its pathogenesis is still poorly understood, and it usually appears during the first few weeks of life and follows a characteristic natural course of proliferation and involution. Most IHs are small, benign, resolve spontaneously, and do not require active treatment but only active observation. A minority of IHs are potentially problematic because they can cause life-threatening complications, permanent disfigurement, and functional impairment. Diagnosis is usually clinical, and propranolol is currently the mainstay of treatment. Other therapeutic modalities may be used alone or in combination, depending on the characteristics of the specific IH. New treatment options are being explored every day, and some are showing promising results. It is undeniable that therapeutic modalities for IHs must be selected based on the child’s age, the size and location of the lesion, the presence of complications, the implementation conditions, and the possible outcomes of the treatment. The future of IH management will certainly be reflected in improved advanced imaging modalities, research into the genetic and molecular basis, the development of new pharmacological agents or techniques, and the development of standardized protocols, all to optimize outcomes with minimal side effects.

## 1. Introduction

Infantile hemangioma (IH) is the most common pediatric benign vascular tumor, with a prevalence of 4–5% [1]. According to ISSVA (the International Society for the Study of Vascular Anomalies) classification, benign vascular tumors are divided as shown in Table 1 [2]. Variant terms such as “capillary hemangioma”, “juvenile hemangioma”, “strawberry hemangioma”, “strawberry birthmark”, or “strawberry nevus” are sometimes used for IH. It can appear anywhere on the body, but the most common localization is on the head (especially the face) and neck [3,4,5]. Regarding the pattern, they can be focal, multifocal, segmental, and indeterminate, while regarding the type, they can be superficial, deep, mixed (superficial + deep), and reticular/abortive/minimal growth. They are also part of PHACE(S) association/syndrome, and LUMBAR association/syndrome [2]. Risk factors include preterm premature rupture of membranes (PPROM), anemia in pregnancy, placenta previa, threatened miscarriage, premature rupture of membranes (PROM), abnormal amniotic fluid volume, prematurity, low birth weight, multiple gestations, female sex, Caucasian heritage, progesterone therapy, and family and miscarriage history [6,7]. It usually appears during the first few weeks of life and follows a characteristic natural course through the phases of proliferation and involution [8]. They should be distinguished from vascular malformations, which are structural anomalies (originating from capillaries, arteries, veins, lymphatic vessels, or a combination thereof, growing proportionally with the child, but generally not regressing), and congenital hemangiomas, which are fully developed at birth and often detected prenatally by ultrasound [2,3,5,9,10]. This comprehensive review aims to present the latest knowledge related to the pathogenesis, clinical characteristics, diagnosis, and management of IHs.

## 2. Pathogenesis

The pathogenesis of IHs has not been fully elucidated to date. IHs are thought to be caused by the “intrinsic abnormal activation” of endothelial cells (ECs) leading to local clonal expansion [11]. The theory that hemangiomas are clonal expansions of cells originating from embolized placental cells or bone marrow cells is reflected in the fact that the pattern of expression of cellular markers (e.g., GLUT-1, merosin, FcRII, Lewis Y antigen, type 3 iodothyronine deiodinase, indoleamine 2,3-deoxygenase, IGF2) and transcriptomes in infantile hemangioma tissue resembles those of ECs lining fetal microvessels in the human placenta [12,13,14,15]. Strub et al. reported that the chromosome 19 miRNA cluster (C19MC), expressed in the placenta, is also expressed in IH ECs. C19MC miRNA levels were found to be elevated only in the circulation of patients with IHs. Circulating miRNA C19MC levels showed a correlation with tumor size and clinical response to oral propranolol, emerging as the first potential biomarker for IH [16]. Also, multiple lines of evidence suggest that in utero hypoxia or local hypoxia may be initiating factors for the development of hemangiomas, with the proliferation phase being a homeostatic attempt to normalize hypoxic tissue [17,18]. Thus, skin sites presenting with IHs may show a precursor lesion, typically manifested as localized pallor, dilated capillaries, and dark red patches, and GLUT-1 was shown to be an important sensor for hypoxia signaling [19,20]. In addition to hypoxia, studies suggest that the renin–angiotensin system (RAS) is involved in the development of IHs through vasoactive peptide angiotensin II (ATII) and considered a key regulator of the hemogenic endothelium via VEGF system and osteoprotegerin, a pro-tumor survival factor, maintaining an environment favorable for vasculogenesis and anti-apoptosis [21,22]. Notably, serum renin levels during infancy and childhood are comparable to the natural history of IHs, as is the efficacy of β-blockers that inhibit renin release, resulting in an accelerated involution process [23,24,25]. Furthermore, it is thought that higher levels of estrogen in pregnant women may affect the vascularization process in the fetus, leading to abnormal vascular proliferation. Of these hormones, 17β-estradiol plays a major role, suggesting that estrogen may be involved in the development of IH. In addition, abnormally high levels of estrogen receptors are found in the tissue structure of IH. The effect of estrogen on angiogenesis is thought to be associated with the expression of angiogenic factors such as VEGF, ultimately leading to abnormal angiogenesis and IH [20,26,27]. It was also found that in some inflammatory environments, inflammatory factors can synergize with other cytokines to promote neovascularization, and a severe imbalance of these factors can lead to the development of IH [28]. The roles played by subpopulations of cells, such as macrophages, mast cells, pericytes, and telocytes, require further study, as they may be closely related to various vascular factors involved in the mechanisms of IH development [20]. A full understanding of the underlying mechanisms would open the door to targeted therapies. The development and establishment of stable and reliable IH models would provide a standardized experimental platform to elucidate its pathogenesis [29].

As we have already mentioned, the course of IHs consists of a proliferative and involutive phase [30]. They usually appear within the first few weeks of life, and the early proliferative phase, when the growth of IHs is fastest, takes place during the first 3–5 months, with peak growth between weeks 5 and 8 (at this stage, IHs usually reach up to 80% of their maximum size) [31,32,33]. The late proliferative phase, in which growth is significantly slower, usually lasts until 9–12 months of age, but growth can exceptionally extend beyond 3 years in large, segmental, and/or deep IHs [34]. The involution phase usually starts from the first year of age and usually lasts 3 to 9 years, leaving fibrofatty residuum in up to 70% of patients [35,36,37].

## 3. Clinical Characteristics

**Figure 1:**
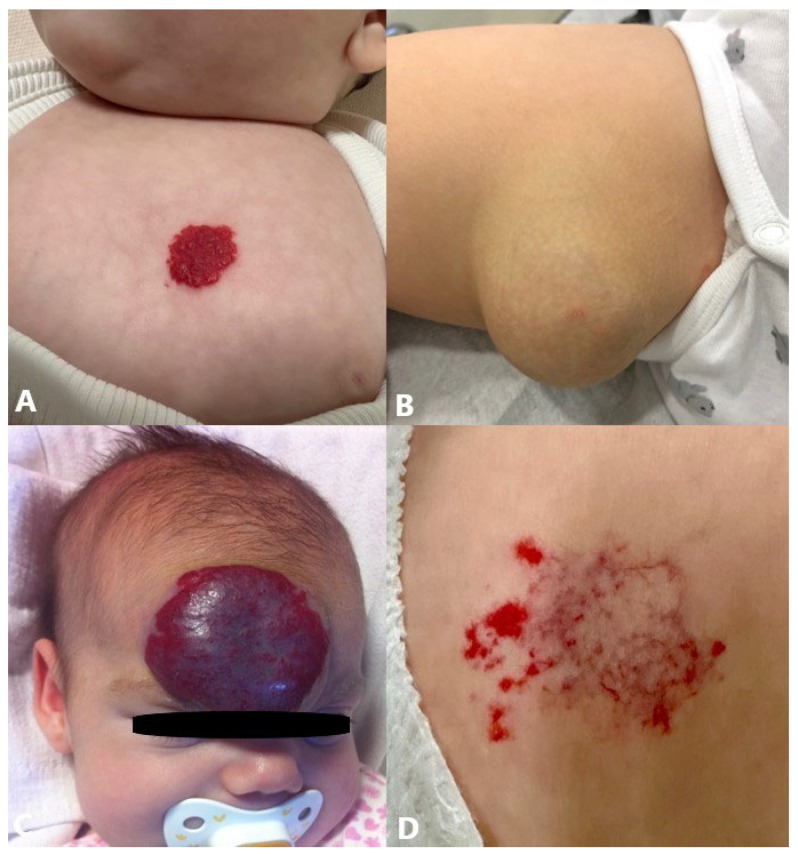
Classification of IHs according to depth: (**A**) superficial IH on chest; (**B**) deep IH on inner right thigh; (**C**) mixed IH at head; (**D**) IH-MAG on abdominal wall.

IHs are clinically classified according to depth into superficial, deep, mixed (superficial + deep), and minimal or arrested growth (IH-MAG) types. They can vary in size from a few millimeters to a few centimeters in diameter. Superficial IHs are the most common type and consist of a bright red papule, nodule, or plaque raised above clinically normal skin (Figure 1A) [38,39]. Deep or subcutaneous IHs involve the deep dermis and/or subcutaneous tissue, presenting as a raised skin-colored nodule, often bluish with or without a central telangiectatic patch (Figure 1B) [8,40]. Mixed IHs are quite common and contain superficial and deep components, that is, they affect the superficial and deep dermis (Figure 1C) [40,41]. IH-MAG usually manifests as a pink blotch, with vasoconstricted areas and/or a perilesional blanching halo, fine or coarse telangiectasia and, when a proliferative phase occurs, bright red papules mostly located in the periphery (Figure 1D) [42,43,44].

**Figure 2:**
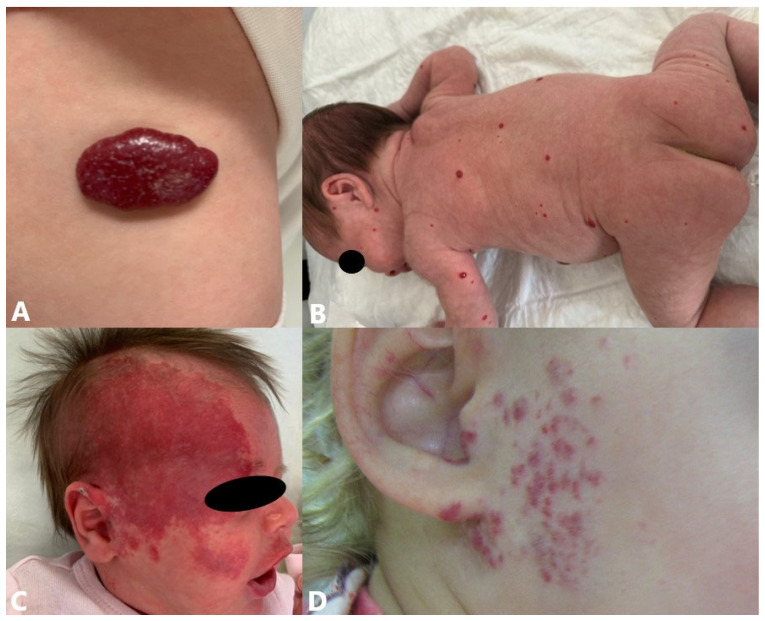
Classification of IHs regarding pattern: (**A**) focal IH on back; (**B**) multifocal IHs; (**C**) segmental IH of frontotemporal region of head; (**D**) indeterminate hemangioma involving preauricular region within boundaries of mandibular segment.

Regarding the pattern, IHs are classified into focal (localized), multifocal, segmental, and indeterminate. Most IHs are focal IHs that are usually small and localized, with a well-defined spatial limitation, and appear to arise from a single focus (Figure 2A) [2,41]. Multifocal IHs are defined as focal lesions affecting more than one anatomic site on the skin (Figure 2B). The multifocal form is a rare form with an increased risk of extracutaneous lesions [45,46]. Segmental IHs are usually patch- or plaque-like with a linear and/or geographic distribution, covering larger anatomical regions, following patterns corresponding to developmental units or anatomical territory supplied by variant embryonic arteries. This form is usually more often associated with complications and generally requires more intensive and long-term therapy (Figure 2C) [2,41,47,48]. Indeterminate IHs are not clearly localized or segmental and are often called partial segmental. Indeterminate hemangiomas lack the round or ovoid shape of many localized hemangiomas yet are smaller than classic segmental lesions and fail to encompass most of a segmental unit, as defined by IH segment maps (Figure 2D) [49].

**Figure 3:**
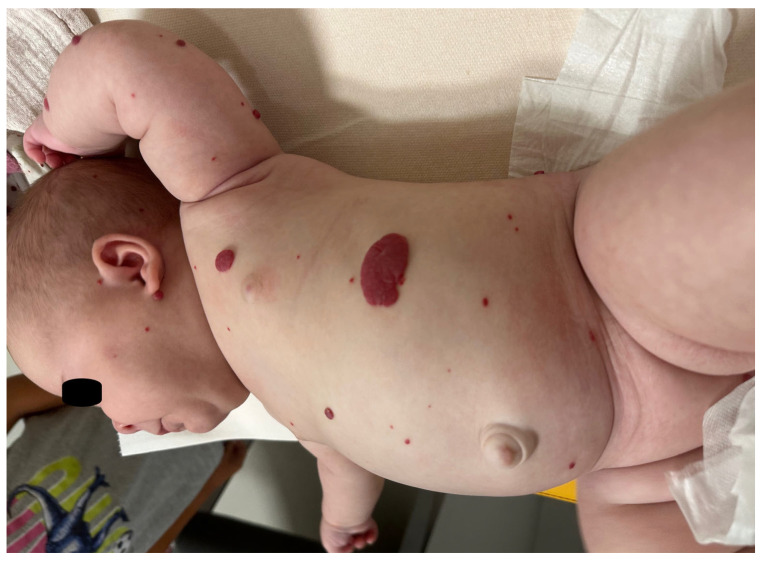
Multiple IHs within IHHs.

Forms of extracutaneous involvement are primarily related to infantile hepatic hemangiomas (IHHs), which are the most common benign liver tumors in infancy [50]. Although it is not the rule, multifocal hepatic hemangiomas are usually present in patients with five or more cutaneous hemangiomas. They are generally asymptomatic, but they can be symptomatic if there are shunts between multiple hemangiomas, or if there is a diffuse hemangioma of the liver when massive hepatomegaly is present (Figure 3) [51,52,53].

**Figure 4:**
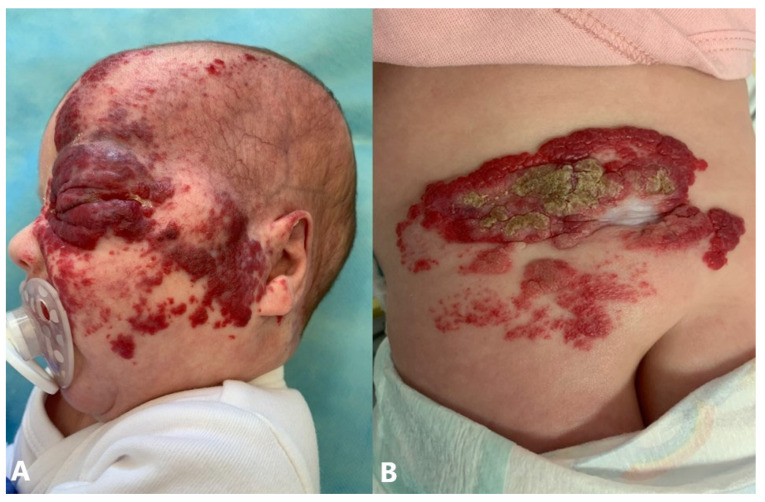
Syndromes associated with segmental IHs: (**A**) large facial segmental IH associated with PHACE(S) syndrome; (**B**) large lumbosacral segmental IH associated with LUMBAR syndrome.

Syndromes associated with segmental IHs are PHACE(S) and LUMBAR syndrome [54]. PHACE(S) syndrome (posterior fossa malformations, hemangioma, arterial anomalies, cardiovascular anomalies, eye anomalies, sternal clefting, and/or supraumbilical raphe) is a neurovascular syndrome defined by the presence of a large segmental IH on the face (may include one to several facial dermatomes) in combination with one or more congenital malformations (Figure 4A) [55,56,57]. PHACE(S) syndrome is observed in 2% to 3% of cases of IH. It is considered one of the most common neurocutaneous vascular disorders in childhood and affects girls more often (9:1). The most common extracutaneous involvement is cerebrovascular therefore neurological and cognitive impairments are potential causes of morbidity in these patients [58]. LUMBAR syndrome (lower body hemangioma, urogenital anomalies, ulceration, myelopathy, bony deformities, anorectal malformations, arterial anomalies, renal anomalies) is characteristically presented by large segmental IH in the lumbosacral area and/or perineum, which often spreads towards one lower extremity (Figure 4B) [59,60,61]. Unlike PHACE(S) syndrome, little is known about the nature of the disease and outcomes of individuals with LUMBAR syndrome. The diagnosis of LUMBAR requires the presence of a segmental, or patterned, IH of the lumbosacral, sacrococcygeal, or pelvic cutaneous regions plus one additional criterion of the urogenital, spinal, bony, anorectal, arterial, or renal organ systems [62].

### Complications

**Figure 5:**
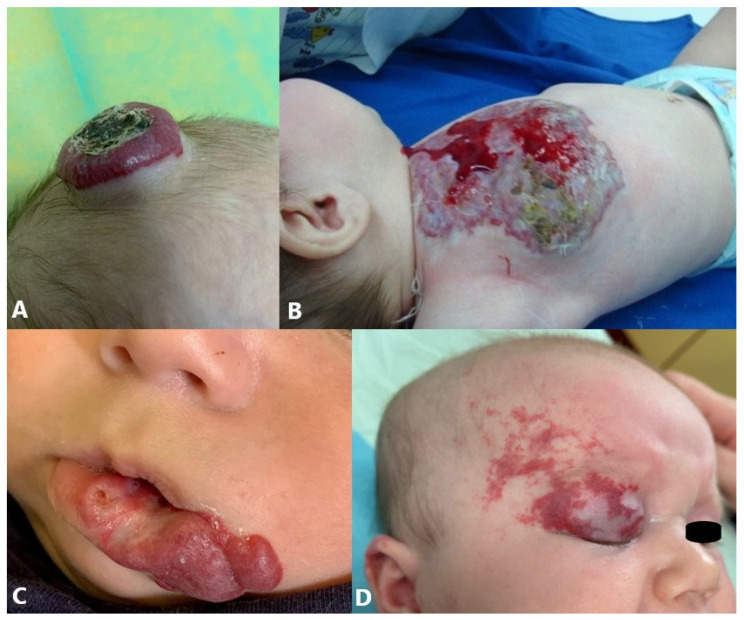
Complications of IHs: (**A**) ulcerated IH on scalp; (**B**) bleeding IH of chest; (**C**) IH of lower lip, gums, and buccal mucosa; (**D**) periorbital IH.

Although most IHs do not present with complications, a certain number develop complications primarily in the form of ulceration, obstruction, functional impairment, and disfigurement. Ulcerations, as the most common form of complication, occur in 11–23% of patients at approximately 2–3 months after birth, but sometimes as early as the neonatal period (Figure 5A) [63,64]. They are especially common when IHs proliferate rapidly or if IHs are present in places subject to trauma or pressure. Factors associated with ulceration include prematurity, female gender, partial segmental morphology, location in the diaper area, and size greater than 5 cm. Complete healing of ulcerated IH usually requires about 3 months. Twelve percent of cases of ulcerated IH treated with propranolol experienced a relapse after discontinuation of treatment. Cases of ulcerated IHs treated with a topical β-blocker did not relapse up to 18 months of follow-up after discontinuation of therapy [64,65]. They are often painful and can lead to bleeding, infection, and scarring [66]. Bleeding is rarely profuse and can generally be stopped by applying direct pressure (Figure 5B). Life-threatening bleeding is extremely rare. Most have occurred in large, bulky mixed- type IHs [67]. Obstruction or functional impairment can be caused by IHs at specific sites. Hemangiomas located along the “beard area” (preauricular region, mandible, lower lip, chin, and anterior neck) can cause airway obstruction, with the most common presentation in the form of hoarseness or stridor at the age of 6–12 weeks when hemangioma proliferation is fastest (Figure 5C) [68,69]. Airway hemangiomas, particularly those causing airway obstruction, account for approximately 1.4% of IH. Upper airway IH often presents with respiratory distress and biphasic stridor, whereas the incidence of pulmonary and lower airway IH is low and was only described in limited cases. Airway obstruction results from IH infiltration of an intraluminal or extraluminal bronchus, causing compression and hyperinflation of the affected side, leading to acute respiratory failure. Such cases require multidisciplinary treatment in intensive care units [70]. Periorbital IHs can induce astigmatism, visual axis obstruction, nasolacrimal duct obstruction, ptosis, amblyopia, and strabismus (Figure 5D). Subcutaneous hemangiomas of the mentioned region are of particular importance because they can extend deep into the orbit, causing exophthalmos or displacement of the globe with only subtle skin manifestations [71,72,73]. Lesions affecting the external acoustic meatus are at risk of conductive hearing loss [74]. Multifocal IHHs can have large vessel shunts that result in heart failure, while in the cases of diffuse IHHs, they can cause impaired ventilation, abdominal compartment syndrome, subsequent multiple organ system failure, and severe hypothyroidism [50,75]. Other rare potential complications of visceral hemangiomas, dependent on specific organ involvement, include gastrointestinal hemorrhage, obstructive jaundice, and central nervous system sequelae due to mass effects [76]. Cosmetic sequelae are significant in approximately half of the untreated IHs and include telangiectasia, fibrofatty tissue, anetoderma, redundant skin, and scarring [77,78].

## 4. Diagnosis

In most cases, the diagnosis of infantile hemangioma can be established clinically, based on history and physical examination [79]. In case of doubt in establishing the diagnosis, especially in the case of segmental lesions, the child should be referred to an appropriately experienced specialist dealing with vascular anomalies. Early referral within 4–6 weeks is of particular importance for infants with high-risk forms of hemangioma who are potential candidates for systemic therapy [53]. Deeper subcutaneous lesions without characteristic skin changes and liver lesions in the absence of skin hemangiomas can be challenging to distinguish from vascular malformations or other vascular tumors. In the mentioned cases, it is necessary to additionally include radiological and pathohistological diagnostics [80,81]. High-frequency ultrasound (HFUS) can be particularly useful for the differential diagnosis of deep IHs, as well as for monitoring the dynamics during the treatment of IHs. Sonographic features will primarily depend on whether the hemangioma is in the proliferative (a well-circumscribed, predominantly hypoechoic, hypervascular tumor with rapid flow) or the involution phase (increased echogenicity and decreased vessel density and size) [82,83]. Computed tomography (CT) is a less commonly used imaging modality because of the increased risk associated with radiation exposure in infants. CT usually shows a well-circumscribed tumor with lobular architecture, and intense homogeneous enhancement. Magnetic resonance (MR) is generally reserved for patients requiring evaluation of associated anatomic anomalies within the syndrome [84,85,86,87]. A biopsy is rarely indicated, and endothelial GLUT-1 staining is a sensitive marker for IH, which is absent in most other vascular tumors and malformations [88,89,90]. Recently, in order to distinguish IHs from other forms of vascular tumors and malformations, electronic colorimeters, widely available on computers and mobile devices, are increasingly being used [91,92].

Despite the updated classification, misdiagnosis remains a major obstacle to optimal assessment and treatment. While infantile hemangiomas have a predictable life cycle, other vascular tumors vary in their onset and growth pattern. Vascular malformations are classified according to the type of vessel and the velocity of blood flow through the vessel, and include capillary, venous, lymphatic, arteriovenous, and mixed malformations. Unlike infantile hemangiomas, vascular malformations are often fully present at birth and grow proportionately with the patient [2,93]. Unlike IHs, congenital hemangiomas appear as mature tumors at birth, and do not proliferate during the first few weeks to months of life. They are usually solitary tumors located on the head, neck, or limbs. Congenital hemangiomas appear as violaceous, pink, or gray rounded plaques with distinct peripheral pallor. They do not express the placental marker, GLUT-1, which is characteristic of IH [94,95]. Kaposiform hemangioendotheliomas (KHEs) are rare, locally aggressive vascular tumors that can present as congenital tumors, but more commonly occur in the first few years of life. They are usually found on the trunk and extremities, and less commonly on the head and neck. KHEs often appear as a flat, vascular patch that becomes progressively indurated. During the rapid growth phase, patients with KHEs are at risk for Kasabach–Merritt phenomenon (KMP) (rapidly growing vascular tumor, thrombocytopenia, consumptive coagulopathy, hypofibrinogenemia), which can lead to significant morbidity and mortality [96]. The histopathological features of tufted angiomas (TAs) and KHEs overlap in some cases, suggesting that the two entities may exist as part of the same spectrum. An indurated, multifocal lesion with overlying hypertrichosis or hyperhidrosis suggests the diagnosis of TA. These tumors should also be followed closely for signs and symptoms of KMP [97]. Pyogenic granulomas (PGs), uncommon in early infancy, appear as bright red, friable, polypoid, or pedunculated lesions often surrounded at the base by a collarette of hyperkeratotic skin that may grow rapidly and bleed profusely at times [95,98]. Capillary malformations (CMs) tend to be light pink at birth and become more red or violaceous over time; they can also be confluent or reticulated. With age, they may also become thicker, indurated, and occasionally hyperkeratotic. Venous malformations (VMs) are uncommon vascular malformations that are present at birth but may not become noticeable until later as the patient grows, do not undergo rapid proliferation over the first several months, and do not involute, as seen with IH. Lymphatic malformations (LMs) are divided into macrocystic, microcystic, and mixed types based on the size of the cysts, and similar to other vascular malformations, LMs tend to progress over time and can cause significant functional impairment [95,99,100,101].

## 5. Management

Guidelines for the approach to the treatment of IHs vary minimally, with the clear intention that the approach to treatment of IH should be individualized, based upon the size of the lesion(s), morphology, location, presence or possibility of complications, potential for scarring or disfigurement, the age of the patient, and the rate of growth or involution at the time of the evaluation [40,53,102,103,104,105,106]. All infants with hemangiomas that are highly suspected of requiring treatment should be referred to a vascular anomaly specialist as soon as possible, preferably by the fifth week of age. Infantile Hemangioma Referral Score can help primary care physicians in this regard [32,107]. Scoring systems were constructed for the management of IHs. The Hemangioma Severity Scale (HSS), which helps decide which IH requires treatment, and the Hemangioma Activity Score (HAS), which serves to monitor therapeutic response, provide a standardized measurement of disease burden based on clinical findings, and represent useful research tools [108,109,110,111]. It is also important to emphasize the psychosocial aspects of care and that providing families with emotional support and reassurance is crucial for the successful treatment of IHs [112,113]. The goals of treatment for hemangiomas must include prevention or reversal of life-threatening or function-threatening complications, prevention or minimization of disfigurement from residual skin changes, minimization of psychosocial distress for the patient and family, and adequate treatment of ulceration to minimize scarring, bleeding, infection, and pain [40]. Medical therapies for hemangiomas are most effective when initiated as early in the hemangioma proliferative phase as possible, ideally within the first two to three months after birth [114]. Below we present the therapeutic options of modern medicine.

### 5.1. Beta-Blockers

#### 5.1.1. Propranolol

In the absence of contraindications, propranolol, a nonselective beta-blocker, is the first-line treatment for high-risk/complicated hemangiomas including large hemangiomas with increased risk of scarring or disfigurement, life-threatening hemangiomas, hemangiomas that pose functional risks, and ulcerated hemangiomas that are unresponsive to standard care [115,116]. In 2008, the incidental observation that the use of propranolol for the treatment of heart failure in two young children with IH resulted in discoloration, softening, and reduction in the size of hemangiomas was a trigger for further research [117,118,119,120,121,122]. Six years later, the US Food and Drug Administration approved an oral solution of propranolol hydrochloride for the treatment of proliferative IH requiring systemic therapy [123].

Patients considering propranolol therapy should undergo a thorough evaluation before treatment. The evaluation includes a detailed medical history with a focus on cardiovascular and respiratory abnormalities, as well as a family history of heart block or arrhythmia. A physical examination includes cardiac and pulmonary assessments, as well as measurements of vital signs, heart rate, and blood pressure [103]. An electrocardiogram is indicated in infants with a history of loss of consciousness, a history of arrhythmias or arrhythmias detected on examination, a lower heart rate, and a family history of congenital heart disease or a maternal history of connective tissue disease [124]. Echocardiography should be considered in infants with an abnormal heart rate, heart murmur, or segmental IH [103]. In children with large, segmental hemangiomas of the head or neck who are at risk for PHACE(S) syndrome; imaging studies, including cardiac ultrasound and MRI of the head/neck and heart, should be performed to exclude serious cardiovascular anomalies [125]. Propranolol treatment is contraindicated in infants and children with cardiogenic shock, chronic and significant sinus bradycardia, chronic and significant hypotension, heart block greater than first degree, heart failure, bronchospasm, hypersensitivity to propranolol, and preterm infants with corrected age <5 weeks (postnatal age in weeks minus number of weeks preterm). Patients with hypoglycemic episodes, wheezing, blood pressure, or heart rate outside the normal range or PHACE(S) syndrome require special caution [103,104].

**Figure 6:**
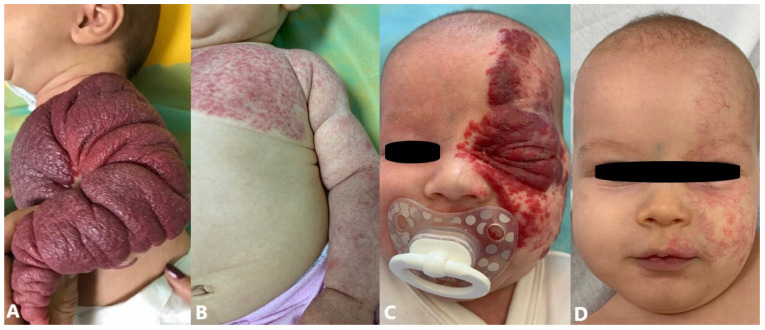
Therapy of IHs with oral propranolol: (**A**,**B**) therapy with propranolol 3 mg/kg/day for 8 months (before and after); (**C**,**D**) therapy with propranolol 2 mg/kg/day for 10 months (before and after).

In healthy infants, treatment can be initiated in an outpatient clinical setting, while in infants <4 weeks of age, with inadequate social support, with comorbid conditions affecting the cardiovascular or respiratory system, including symptomatic airway hemangiomas, and with conditions affecting blood glucose maintenance, hospitalization is recommended [116,124,126,127]. Treatment is initiated with oral propranolol, immediately after feedings to reduce the risk of hypoglycemia, at a dose of 1 mg/kg/day in two divided doses, increasing the dose to 2 mg/kg/day in two divided doses after one week, and then to 3 mg/kg/day in two divided doses, depending on the severity of the hemangioma and the clinical response (Figure 6A,B) [103,104,105,128]. In PHACE(S) syndrome, starting propranolol at a lower dose of 0.5 mg/kg/day in three doses instead of two may be considered (Figure 6C,D) [129]. The effect of oral propranolol peaks one to three hours after administration, and during this interval, special attention should be paid to possible hypotension, bradycardia, wheezing, and hypoglycemia [130]. With regular check-ups to assess response and adjust the dose for weight gain, propranolol treatment is usually discontinued at 12–18 months of age but may last longer in some patients [131,132]. Rebound growth, particularly when therapy is discontinued before 9 months of age, in female gender, head and neck location, segmental pattern, and deep involvement, is relatively common (19–25%) after cessation of therapy, and a minority of IH are resistant to propranolol. The estimated incidence of propranolol-resistant IH is <1%, defined as continued growth during the proliferative phase or no reduction in size during the post-proliferative phase, after at least 4 weeks of oral propranolol at a dose of ≥2 mg/kg/day [133,134,135].

In patients with small superficial hemangiomas, topical propranolol is available in the form of creams, unguents, and gels prepared with galenical formulations with concentrations ranging from 0.5% to 5% [78,136]. So far, only minor local side effects were observed, including irritation, redness, and scaling of the treated area, with no systemic side effects [137].

#### 5.1.2. Timolol

**Figure 7:**
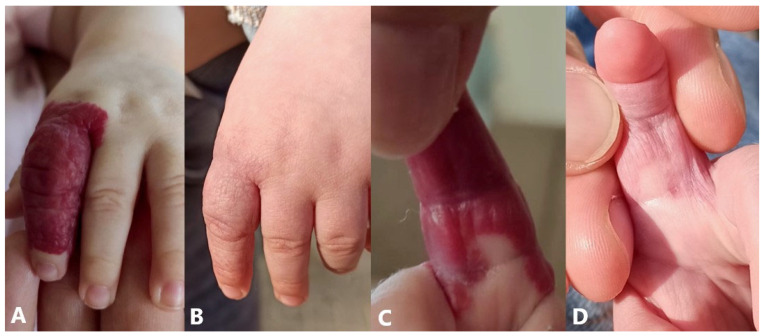
Topical therapy with 0.5% timolol maleate solution for 8 months: (**A**,**B**) dorsal side of index finger (before and after); (**C**,**D**) volar side of index finger (before and after).

Topical timolol, as a non-selective beta-blocker, was initiated in the treatment of thin (<2 mm in thickness) superficial hemangiomas, as well as in the treatment of minor ulcerations and to minimize rebound growth in children treated with oral propranolol. Treatment consists of 0.5% timolol maleate solution, one to two drops two to three times daily until the patient is 12 months old or until satisfactory improvement is achieved (Figure 7) [138,139]. Topical timolol is generally well tolerated, with minimal adverse effects such as eczema, local irritation, ulceration, skin rashes, desquamation, erythema, and bronchospasm, usually without the need for discontinuation of therapy [140,141].

#### 5.1.3. Other Beta-Blockers

According to the studies available so far, the use of atenolol as a hydrophilic selective β1-blocker, at a dose of 1 mg/kg per day, is safe and effective. Compared to propranolol, it had comparable efficacy with fewer side effects (no significant hypoglycemia, bronchospasm, bradycardia, or hypotension occurred) [142,143,144].

Nadolol, as a nonselective beta-blocker, showed better mean involution size and mean color fading compared to propranolol in increasing doses up to 2 mg/kg/day. Side effects occurred with a similar frequency compared to propranolol and included mainly upper respiratory tract infections, diarrhea, vomiting, and sleep disturbances [145,146]. It should be noted that one possible related death in an infant treated with nadolol was reported in the literature [147].

Carteolol, another non-selective beta-blocker, has shown successful topical action, with early administration of which good results can be achieved and functional impairment can be avoided [148].

In subglottic hemangioma, treatment with the cardioselective beta-blocker acebutolol, 8 mg/kg/day, can be successful, without side effects [149].

Further studies are warranted, especially focusing on the pharmacokinetics of these drugs in infants.

### 5.2. Corticosteroids

Before the introduction of beta-blockers in treating IHs, systemic corticosteroids were the mainstay of IHs treatment for many years. However, they are still used today in patients with complicated hemangiomas in whom beta-blocker treatment is contraindicated. They are also combined with beta-blockers to shorten the time to respond in selected cases [150]. They can be administered orally, intravenously, intramuscularly, and topically, but their systemic long-term administration at high doses can lead to various undesirable side effects [151]. The recommended initial dose of prednisolone treatment is 1–3 mg/kg/day, and the duration of treatment ranges from long cycles of 9 to 12 months, with full dose maintenance for 4 to 12 weeks, to shorter cycles of 1 to 6 weeks, with multiple intermittent courses if needed [152]. It can be used as monotherapy or in combination with propranolol, which may accelerate the involution of IH compared to monotherapy, especially in life- or function-threatening IHs that require urgent treatment [150,153,154]. Topical application was generally shown to be safe with a rapid response and is used for small tumors, although topical application can also result in side effects such as skin atrophy, hypopigmentation, and hypertrichosis [155,156]. Intralesional corticosteroids, such as triamcinolone acetonide 10 to 40 mg/mL, are limited to well-localized hemangiomas with an initial response within 2 weeks and continued response over the ensuing 6–8 weeks [157,158,159,160]. Also, the intralesional administration of betamethasone ranging from 0.5 mL to 2 mL according to the tumor area is a feasible choice for small-sized hemangiomas [155,161].

### 5.3. Sirolimus (Rapamycin)

Sirolimus, an mTOR inhibitor, has emerged as an option for the treatment of inoperable IHs that respond inadequately to first-line treatment with propranolol and/or prednisolone [162,163,164]. It was proven that sirolimus reduces the self-renewal of IH stem cells, reduces differentiation, reduces the proliferation of GLUT1+ cells, inhibits vasculogenesis, and facilitates the regression of the established vasculature, but the role and final effectiveness have yet to be further clarified in future research [165,166,167]. Due to adverse effects such as immunosuppression, metabolic disturbances, and potential renal toxicity, therapeutic drug monitoring is required to ensure adequate serum levels [168]. Future research must provide answers about the most appropriate dose and duration of treatment [169,170].

### 5.4. Imiquimod

Imiquimod, an imidazoquinoline amine, with its ability to induce the production of interferon, tumor necrosis factor-alpha, and the antiangiogenesis factor tissue inhibitor of matrix metalloproteinase, in the form of imiquimod 5% cream was an interesting therapeutic alternative that accelerated the involution of superficial IHs [171,172,173,174]. With the arrival of timolol maleate, it showed better effects for color involution, onset time, and side effects than imiquimod [175,176].

### 5.5. Itraconazole

Itraconazole, a systemic triazole drug with potent antiangiogenic properties, was shown in previous studies to be a suitable alternative to oral propranolol. It was even shown to have faster effects, i.e., shorter treatment time, with a similar safety profile including all side events. Given the small sample size of pediatric patients, further analyses are needed to confirm the findings [177,178,179].

### 5.6. Sclerotherapy

Sclerotherapy is a simple, easy, and effective, but still under-studied method for treating IHs. The sclerosant disrupts cell membranes, causing thrombosis, obliteration of the vascular lumen, and irreversible damage to the vascular endothelium. Sclerosing agents tried in IH include polidocanol, sodium tetradecyl sulfate, bleomycin, ethanolamine oleate, etc., with the indication that polidocanol is the most commonly used given its additional anesthetic effect, low toxicity, and low risk of allergic reactions [180,181,182].

If pharmacotherapy does not have a satisfactory effect, laser therapy or surgical treatment may be recommended.

### 5.7. Laser Therapy

**Figure 8:**
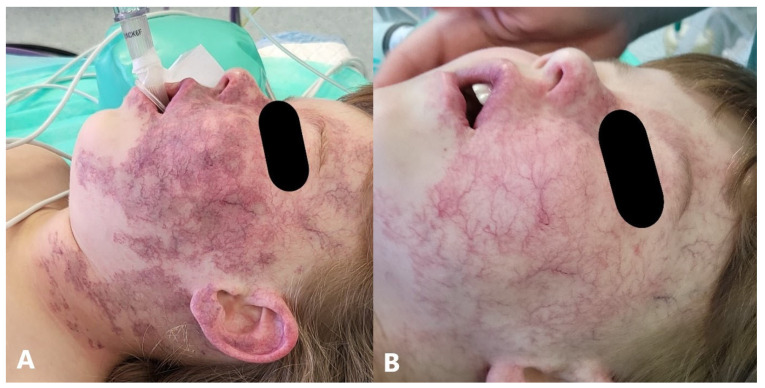
PHACE(S) syndrome: (**A**) residual IH after completion of propranolol therapy; (**B**) after PDL laser treatment.

Pulsed dye lasers (PDLs) are the most common lasers used to treat IHs. Laser therapy may be beneficial in patients with small, superficial, or ulcerated lesions, as well as postinvolution erythema and telangiectasia, while for larger lesions it can be combined with oral beta-blockers, providing additional treatment benefits [183,184,185,186]. In addition to the combination with oral propranolol, PDL has also shown excellent results in combination with topical timolol [187,188]. The ideal timing of PDL therapy in IHs remains uncertain, but it was established that involuting hemangiomas respond better than actively proliferating lesions, and thus PDL may be most useful for accelerating the clinical course of actively involuting lesions [189]. Commonly used parameters for PDL in the treatment of infantile hemangiomas are as follows: wavelength—585 to 595 nm; fluence—5 to 7.5 J/cm^2^ (low fluences should be used for proliferating lesions, and lesions in areas with thin skin); pulse duration—0.45 to 6 ms; spot size—5 to 12 mm. Treatments are usually performed at intervals of two to four weeks for rapidly proliferating or ulcerated lesions, while stable or involuting lesions can be treated less frequently, every four to six weeks (Figure 8) [190,191]. Reported side effects of PDL use can include swelling, hyperpigmentation or hypopigmentation, pain, ulceration, scarring, and bleeding [192]. In addition to PDL, the Nd:YAG laser has proven to be preferable for deeper forms, while the combination of PDL and Nd:YAG has proven to be an option for mixed type IH [193,194]. Ablative fractionated carbon dioxide laser and Er:YAG laser were also used to assist in the transdermal delivery of timolol to deep IH with fully ablative carbon dioxide lasers employed in subglottic IH [195,196].

### 5.8. Surgery

**Figure 9:**
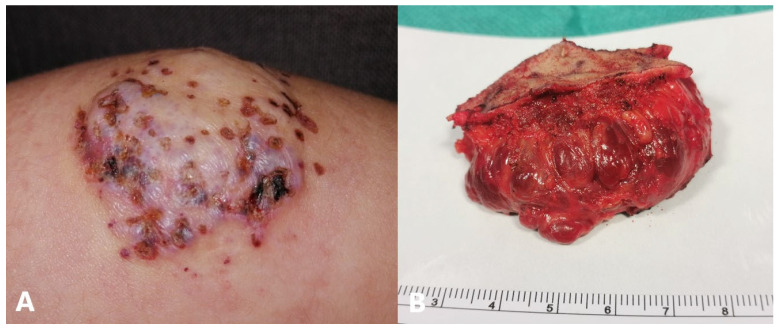
Mixed type of IH of upper leg: (**A**) clinical before excision; (**B**) excision of IH (5 × 3 cm).

Surgical treatment usually applies to involuted lesions with residual scarring or loose skin. Surgery can also be considered for severely ulcerated hemangiomas that are resistant to other therapies and in cases of IHs that directly threaten life or function [197,198]. If there is an indication for surgical treatment, it is best to perform it before the fourth year of life to prevent psychosocial morbidity. Since the tumor usually acts as a natural tissue expander, in most cases, an adequate amount of skin is available for wound closure. Traditional lenticular excision/linear closure is applicable in some locations, such as the scalp, eyelid, lip, and abdomen, while circular excision/purse-string closure should be considered for circular lesions located on the face (Figure 9) [199]. In rare cases of large, visceral (especially hepatic) hemangiomas, and in those complicated by hemorrhage, arterial embolization is the method of choice. Embolization may be considered in lesions that are refractory to medical therapy, cause organ damage or heart failure, are expansive, or are unsuitable for surgery [200,201,202].

## 6. Conclusions

The future of managing IHs will certainly be reflected in improved advanced imaging modalities, investigations of the genetic and molecular basis in the search for diagnostic markers, and a better understanding of the role of various growth factors, signaling pathways, and genetic mutations. Moreover, the development of new pharmacological agents or techniques, such as the development of targeted therapies, and the development of standardized protocols for longitudinal monitoring of IHs to identify IHs that require intervention from those that can be observed, can all aid in optimizing outcomes with minimal side effects.

## Figures and Tables

**Table 1:** ISSVA classification of benign vascular tumors [2].

Benign Vascular Tumors	
Infantile hemangioma	
Congenital hemangioma	Rapidly involuting (RICH) * Non-involuting (NICH) Partially involuting (PICH)
Tufted angioma *^,^ **	
Spindle-cell hemangioma	
Epithelioid hemangioma	
Pyogenic granuloma ***	
Others	Hobnail hemangioma Microvenular hemangioma Anastomosing hemangioma Glomeruloid hemangioma Papillary hemangioma Intravascular papillary endothelial hyperplasia Cutaneous epithelioid angiomatous nodule Acquired elastotic hemangioma Littoral cell hemangioma of the spleen
Related lesions	Eccrine angiomatous hamartoma Reactive angioendotheliomatosis Bacillary angiomatosis

* Some lesions may be associated with thrombocytopenia and/or consumptive coagulopathy. ** Many experts believe that tufted angioma and kaposiform hemangioendothelioma are part of a spectrum rather than distinct entities. *** Also known as lobular capillary hemangioma.

## Data Availability

Not applicable.
